# Characteristics and dynamic changes of gut microbiota in Mongolian horses and Guizhou horses

**DOI:** 10.3389/fmicb.2025.1582821

**Published:** 2025-04-15

**Authors:** Yaonan Li, Yanfang Lan

**Affiliations:** ^1^School of Physical Education and National Equestrian Academy, Wuhan Business University, Wuhan, China; ^2^Research Center for Modern Equine Industry Development, Wuhan, China

**Keywords:** gut microbiota, Guizhou horses, Mongolian horses, diversity, intestinal functions

## Abstract

The gut microbial importance and its crucial roles in host digestion, immunity, and metabolism have received widely attention. Horses, especially indigenous varieties such as Mongolian horses (MGH) and Guizhou horses (GZH), have not received sufficient attention, and the characteristics of their gut microbiota are still unclear. For this purpose, we collected faecal samples from eight MGH and eight GZH to compare their gut microbial differences using amplicon sequencing. The results of alpha diversity analysis indicated that the gut bacterial diversity and gut fungal abundance in GZH were significantly higher than those in MGH. Meanwhile, beta diversity revealed that there were significant differences in the gut bacterial and fungal structures between GZH and MGH. Although the dominant bacterial and fungal phyla of GZH and MGH were the same, there were a large number of significantly different bacteria and fungi between both groups. Moreover, we observed that there were 32 phyla (23 bacterial phyla and 9 fungal phyla) and 718 genera (383 bacterial genera and 335 fungal genera) with significant differences between the GZH and MGH. Notably, this study also revealed some differences in intestinal functions between MGH and GZH, such as chemoheterotrophy, fermentation, and cellulolysis. To our knowledge, this is the first report on the comparative analysis of the gut microbiota between MGH and GZH. Our results demonstrated that GZH have a richer and more diverse gut microbiota compared with MGH. Additionally, these results are important for understanding the gut microbial characteristics of indigenous horse.

## Introduction

It is well-known that the intestine is the main habitat of microorganisms and the main organ for digestion and absorption (Chen C. et al., 2021; Ramirez et al., 2020; Yu et al., 2024). These gut-residing microorganisms, primarily bacteria and fungi, play important roles in host health and intestinal function (Li X. et al., 2023). For instance, surveys have indicated that gut microbiota could produce beneficial metabolites such as short-chain fatty acids, digestive enzymes, vitamins, and antimicrobial peptides, which are beneficial to improve intestinal environment, promote animal growth performance, and prevent pathogenic bacteria infection (Chadchan et al., 2021; Liang et al., 2022). Moreover, several studies involving gut microbiota have also revealed their importance in intestinal barrier function, mucosal immunity, and epithelial cell differentiation (Meng et al., 2024; Schluter et al., 2020; Sun et al., 2022). Previous studies indicated that intestinal homeostasis and intestinal function depends on the stabilized gut microbiota (Yang et al., 2024). However, the gut microbial composition and structure are affected by both host genetics and external factors. External factors such as antibiotics, heavy metals, pesticides, and microplastics could perturb gut microbial homeostasis, inducing gut microbial dysbiosis (Bariod et al., 2025; Hotchkiss et al., 2022). Studies have shown that gut microbial dysbiosis is closely associated with the development of many gastrointestinal diseases including diarrhea, inflammatory bowel disease, and colorectal cancer (Vich et al., 2018; Xie et al., 2024; Zuo et al., 2018). Moreover, gut microbial dysbiosis has also been demonstrated to play an important role in the diabetes, Parkinson’s disease, and obesity (Hattori et al., 2021; Xue et al., 2023). Therefore, exploring the gut microbial composition and structure is crucial to maintaining host health. Notably, the host genetics can also have a significant impact on the gut microbial composition and structure (Wen et al., 2021). Early investigations suggested that different species will evolve significantly different gut microbiota due to the differences in genetic background. Not only that, there are also significant differences in the gut microbiota between different varieties of the same species (Ma et al., 2022).

Numerous studies indicated that the horses are one of the earliest domesticated animals, which played a vital roles in the development of human civilization and society (Jin et al., 2023). In ancient, the horses were primarily used in military, agriculture, and transportation. Nowadays, more horses are predominantly used for recreation and competitive sports (Hanousek et al., 2020). According to statistics, there are approximately 60 million horses worldwide, containing more than 300 different breeds. China is an important horse breeding and producing country with about 30 local breeds including MGH and GZH. MGH, native to the grasslands of Inner Mongolia, China, are characterized by strong adaptability and cold-resistant. On the other hand, GZH are mainly distributed in southwest China, such as Guiyang, Bijie, Liupanshui, with a population of about 350,000. Both MH and GH are traditional local horse breeds in China that play important role in national culture and social development. Previous studies have indicated that there are significant differences in the gut microbiota between different breeds of the same species (He et al., 2022). However, studies regarding the gut microbiota in MGH and GZH remains scarce. Thus, we compared the gut bacterial and fungal compositions and differences between the MGH and GZH by 16S rDNA and ITS2 amplicon sequencing.

## Materials and methods

### Sample acquisition

In this study, 8 GZH and 8 MGH were used as experimental animals. The ratio of females to males in each group is 1:1. We first evaluated the health status of each horse to decrease the influence of other diseases on gut microbiota. Moreover, these selected horses had not been injected with antibiotics recently. Fresh fecal samples were collected from the designated area and the dirt on the surface was removed. Subsequently, the fecal samples were spread and the middle part was collected to reduce contamination and ensure the accuracy of the results. Fecal samples collected from GZH and MGH were individually placed in EP tubes and labeled (MGH: MGH1, MGH2, MGH3, MGH, MGH5, MGH6, MGH7, MGH8; GZH: GZH1, GZH2, GZH3, GZH4, GZH5, GZH6, GZH7, GZH8). Finally, the samples were refrigerated at −80°C for 16S rDNA and ITS2 Amplification Sequencing.

### 16S rDNA and ITS2 amplification sequencing

Microbial DNA was extracted from selected fecal samples from both the MGH and GZH using the QIAamp DNA Mini Kit (QIAGEN, Hilden, Germany) following the manufacturer’s instructions. The purified DNA was then evaluated for quality using a spectrophotometer and agarose gel electrophoresis. Universal primers (338F: ACTCCTACGGGAGGCAGCA and 806R: GGACTACHVGGGTWTCTAAT; ITS5F: GGAAG TAAAAGTCGTAACAAGG and ITS2R: GCTGCGTTCTTCATCGA TGC) were synthesized to amplify the V3/V4 and V3/V4 regions. Three PCR reactions were performed in a 20 μL system based on the previous PCR cycle parameters (Lan et al., 2024). The PCR amplification products were subsequently extracted from a 2% agarose gel, further purified, and quantified. Meanwhile, the target fragments was required to recycle utilizing gel recovery kit (Axygen, CA, USA). The samples from MGH and GZH were mixed according to the fluorescence quantification results and sequencing volume requirements to prepare sequencing libraries. Before sequencing, the libraries underwent additional processing steps, including purification, quality assessment, and quantification. Sequencing libraries were constructed employing PacBio platform (Biomarker Technologies, China). Only libraries meeting the criteria of a single peak and a concentration greater than 2 nM were subjected to 2 × 300 bp paired-end sequencing on a MiSeq sequencer.

### Bioinformatics and statistical analysis

The sequences obtained from 16S rDNA and ITS2 amplicon sequencing need to undergo preliminary screening to remove chimeras, mismatches, low-quality, and short sequences. Initially, quality screening and primer elimination of the original data were conducted to obtain clean reads utilizing Trimmomatic (v0.33) and Cutadapt software (1.9.1). The resulting clean reads were then spliced and subjected to a secondary filtering process as per the length of the spliced sequences, employing Usearch software (v10). Subsequently, chimera sequences in the raw data were identified and removed using UCHIME software (v4.2) to yield effective reads. These sequences are identified to OTUs based on the 97% similarity threshold. Moreover, we also identified the species and abundances of dominant bacteria and fungi at the phylum and genus levels. Prior to the above analysis, rarefaction curves and rank abundance curves were generated to assess the sequencing depth. In order to compare the differences in gut microbial diversity and abundance, several microbial indices such as Chao1, ACE, Shannon, and Simpson are calculated based on the abundance of OTUs in different samples. Meanwhile, beta diversity analysis is conducted by generating PCoA scatter plots to explore shifts in gut microbial structure. Furthermore, Metastats and LEfSe analysis are used to identify taxa with significant differences between the MGH and GZH. Data are presented as mean ± SEM, and statistical significance is determined using a standard threshold of *p* < 0.05.

## Results

### Analysis of sequencing sequence and OTUs number

**Figure 1:**
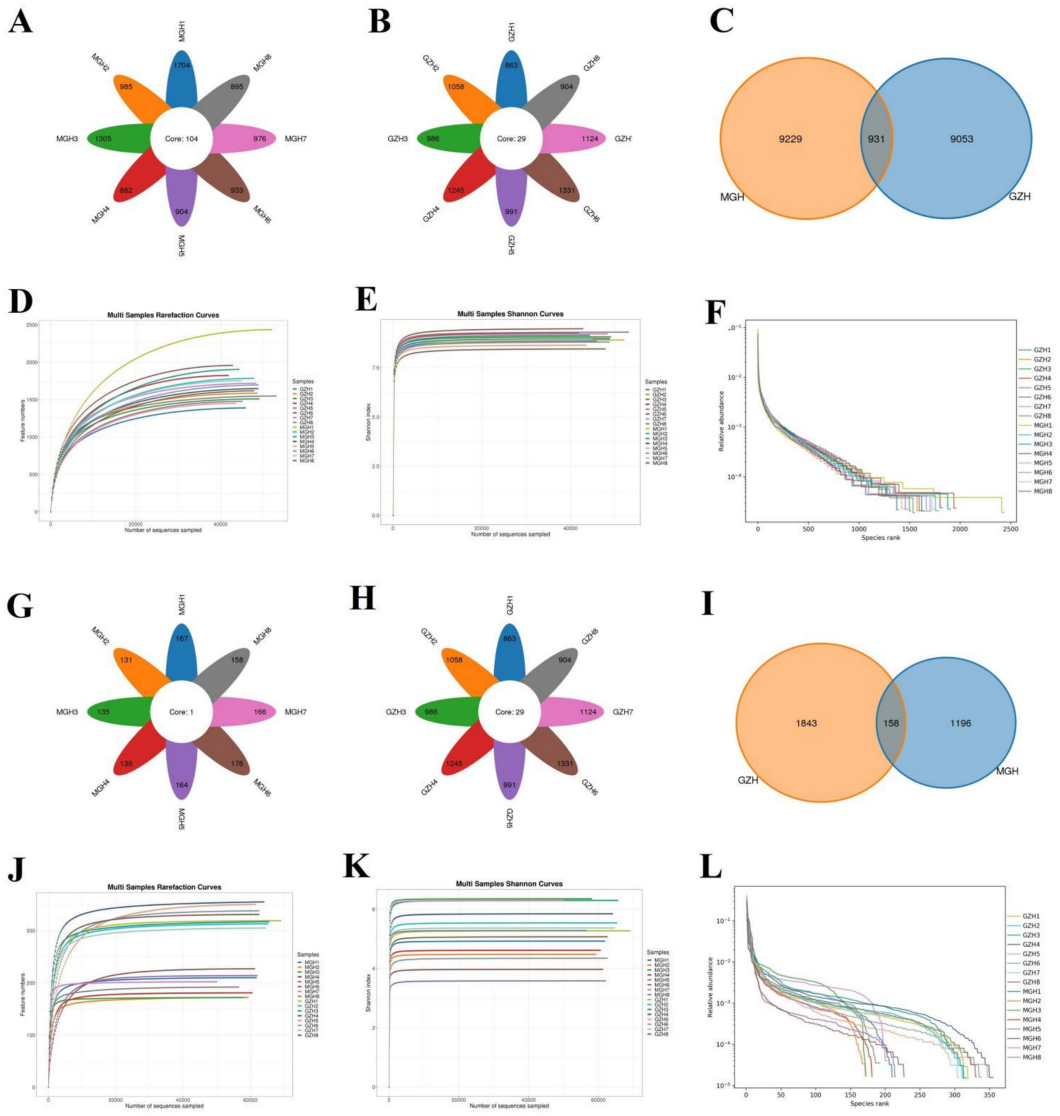
Venn diagram and sequencing depth evaluation. Venn diagram showing the number of common and individual OTUs [**(A–C)** Venn diagram in the gut bacterial community; **(G–I)** Venn diagram in the gut fungal community] in the MGH and GZH. Gut bacterial **(D–F)** and fungal **(J,K,L)** rarefaction curves and rank abundance curves for the samples collected from MGH and GZH.

In this research, 16 fecal samples were collected to compare the differences in gut bacterial and fungal communities between GZH and MGH. Results indicated that 1,279,919 (MGH = 640,188, GZH = 639,731) and 1,277,428 (MGH = 637,530, GZH = 639,898) raw sequences were achieved from the gut bacterial and fungal communities of GZH and MGH, respectively (Tables 1, 2). Moreover, these raw data need to be further processed to obtain reliable valid sequences. After treatment, a total of 761,908 (MGH = 383,128, GZH = 378,780) valid bacterial sequences and 986,390 (MGH = 470,629, GZH = 515,761) valid fungal sequences were collected, with an efficiency of 59.52 and 77.21%, respectively. These valid sequences were subsequently clustered into OTUs based on 97% sequence similarity. Results indicated that a total of 19,213 bacterial OTUs (GZH1 = 1,389, GZH2 = 1,586, GZH3 = 1,510, GZH4 = 1,823, GZH5 = 1,478, GZH6 = 1,957, GZH7 = 1,718, GZH8 = 1,549, MGH1 = 2,435, MGH2 = 1,785, MGH3 = 1,904, MGH4 = 1,647, MGH5 = 1,451, MGH6 = 1,696, MGH7 = 1,752, MGH8 = 1,618) and 3,197 (GZH1 = 319, GZH2 = 313, GZH3 = 317, GZH4 = 355, GZH5 = 350, GZH6 = 338, GZH7 = 305, GZH8 = 331, MGH1 = 210, MGH2 = 172, MGH3 = 172, MGH4 = 181, MGH5 = 214, MGH6 = 227, MGH7 = 202, MGH8 = 192) fungal OTUs were identified (Figures 1A–C,G–I). Moreover, we also observed 931 core bacterial OTUs and 158 core fungal OTUs in GZH and MGH, accounting for 4.85 and 4.94% of the total OTUs number, respectively. The rarefaction curve results show that all curves are close to saturation trends, indicating that further increasing the sequencing depth cannot find more bacterial and fungal taxa (Figures 1D–F,J–L).

**Table 1:** Statistics of bacterial sequence information generated during amplicon sequencing.

Sample ID	Raw reads	Clean reads	Denoised reads	Merged reads	Non-chimeric reads
MGH1	80153	71283	70433	58756	52986
MGH2	80034	72449	71847	57787	48304
MGH3	80020	71386	70579	57710	45557
MGH4	80062	72097	71459	58803	49208
MGH5	79853	71932	71244	57974	44004
MGH6	80154	71706	71145	58478	49288
MGH7	79939	72401	71866	56523	45434
MGH8	79973	72670	72014	59138	48347
GZH1	80127	72399	71931	60026	46447
GZH2	80120	72208	71709	60878	48942
GZH3	79962	71831	71362	61331	49412
GZH4	79926	72487	71834	58305	42388
GZH5	79938	71782	71268	59716	45662
GZH6	79945	73257	72653	58342	43461
GZH7	79988	72554	71966	60134	48891
GZH8	79725	71774	71257	62978	53577

**Table 2:** Statistics of fungal sequence information generated during amplicon sequencing.

Sample ID	Raw reads	Clean reads	Denoised reads	Merged reads	Non-chimeric reads
MGH1	80009	63752	63679	62689	62002
MGH2	80067	65055	64998	60733	59406
MGH3	79971	66694	66657	63035	58137
MGH4	80048	63322	63284	62102	60706
MGH5	80000	64801	64779	62107	62037
MGH6	80091	65232	65169	61498	61382
MGH7	79876	58807	58749	52956	50242
MGH8	77468	61192	61136	58334	56717
GZH1	80109	70071	69974	69512	69141
GZH2	79990	67339	67318	66461	65474
GZH3	80000	68143	68104	67336	65631
GZH4	79809	68139	68056	66990	64028
GZH5	79984	63770	63750	62883	61613
GZH6	80157	63348	63330	62699	62592
GZH7	79677	65981	65957	65399	64618
GZH8	80172	63987	63965	63192	62664

### Comparative analysis of gut bacterial and fungal diversities

**Figure 2:**
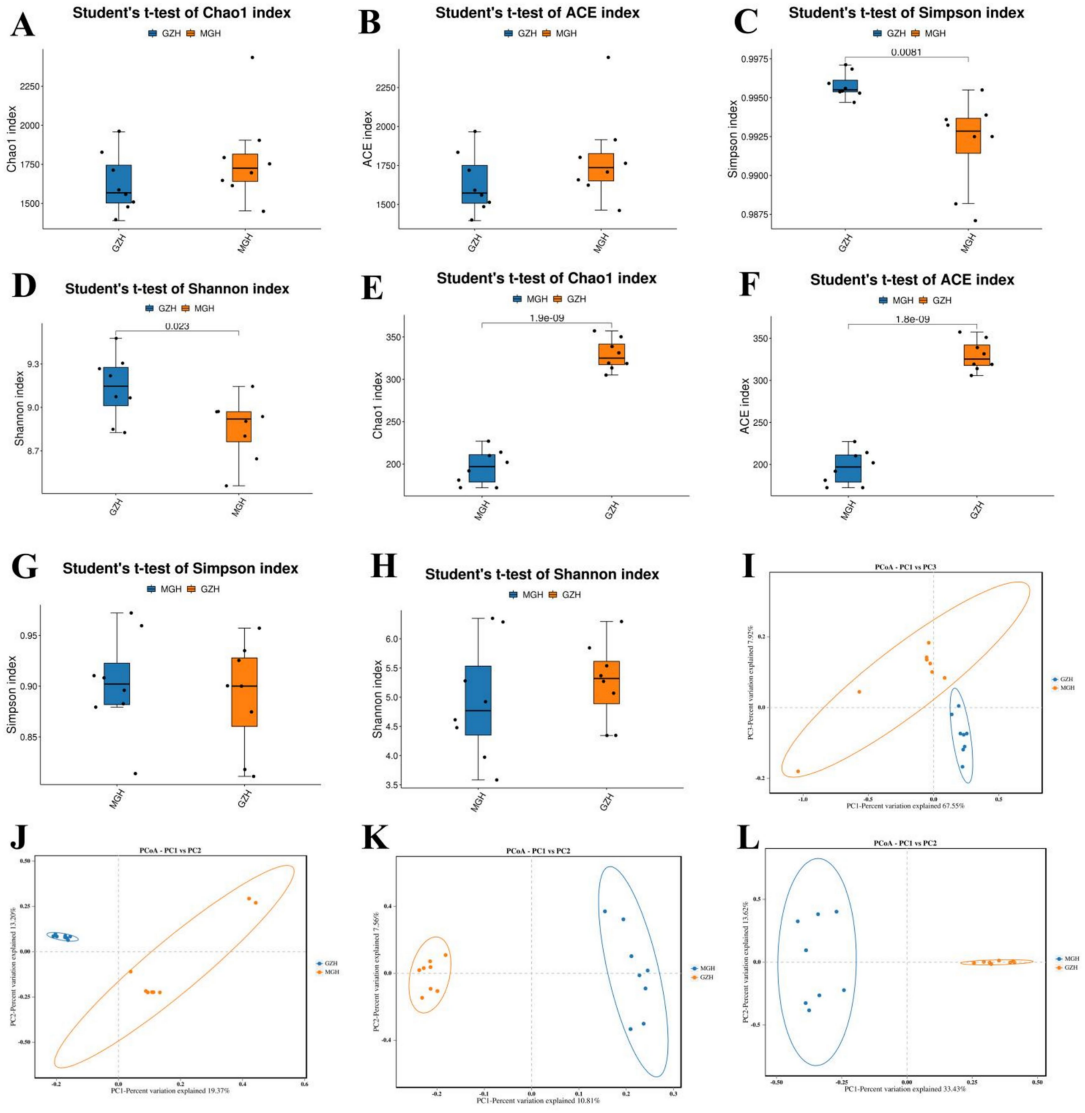
Comparison of gut bacterial and fungal diversities between the MGH and GZH. The diversity (measured by Chao1 and ACE indices) and abundance (measured by Simpson and Shannon indices) of gut bacterial **(A–D)** and fungal **(E–H)** communities in the MGH and GZH. For comparative analysis gut bacterial **(I,J)** and fungal **(K,L)** structures, we drew the PCoA scatter plots.

The average of bacterial Chao1 and ACE indices in GZH were 1,626.65 and 1,632.83, whereas the bacterial Shannon and Simpson indices were 9.13 and 0.99, respectively. Moreover, the above-mentioned four indices in the MGH were 1,786.95, 1,797.29, 8.85, and 0.99, respectively. Intergroup analysis revealed that there were significant differences in the bacterial Simpson (0.99 ± 0.0003 versus 0.99 ± 0.001, *p* = 0.0081) and Shannon (9.13 ± 0.079 versus 8.85 ± 0.076, *p* = 0.023) indices, whereas the Chao1 (1,626.65 ± 67.59 versus 1,786.95 ± 103.77, *p* = 0.21) and ACE (1,632.83 ± 67.86 versus 1,797.29 ± 103.62, *p* = 0.22) indices were not significantly different between the GZH and MGH. The above results showed that the gut bacterial diversity in GZH is significantly higher than that in MGH, whereas the difference in the gut bacterial abundance is not significant (Figures 2A–D). As for the gut fungal community, the average of Chao1, ACE, Shannon and Simpson indices in the GZH were 329.18, 329.63, 5.25 and 0.89, while these indices in the MGH were 196.25, 196.46, 4.93 and 0.90, respectively. Comparative analysis of diversity showed that the Chao1 (329.18 ± 6.49 versus 196.25 ± 7.20, *p* < 0.00000001) and ACE (329.63 ± 6.49 versus 196.46 ± 7.18, *p* < 0.00000001) indices of the GZH were significantly higher than those of the MGH, while the Shannon (5.25 ± 0.23 versus 4.93 ± 0.35, *p* = 0.46) and Simpson (0.89 ± 0.018 versus 0.90 ± 0.017, *p* = 0.62) indices were not significantly different (Figures 2E–H). This indices that GZH have a higher gut fungal abundance as compared to MGH. We also used PCoA plots to further analyze the differences in gut bacterial and fungal communities structures between the GZH and MGH. Results indicated that samples in the same clustered together, but samples in different groups were clearly separated, suggesting the significant difference in the gut bacterial and fungal communities structures between GZH and MGH (Figures 2I–L).

### Analysis of gut bacterial composition and differential taxa

**Figure 3:**
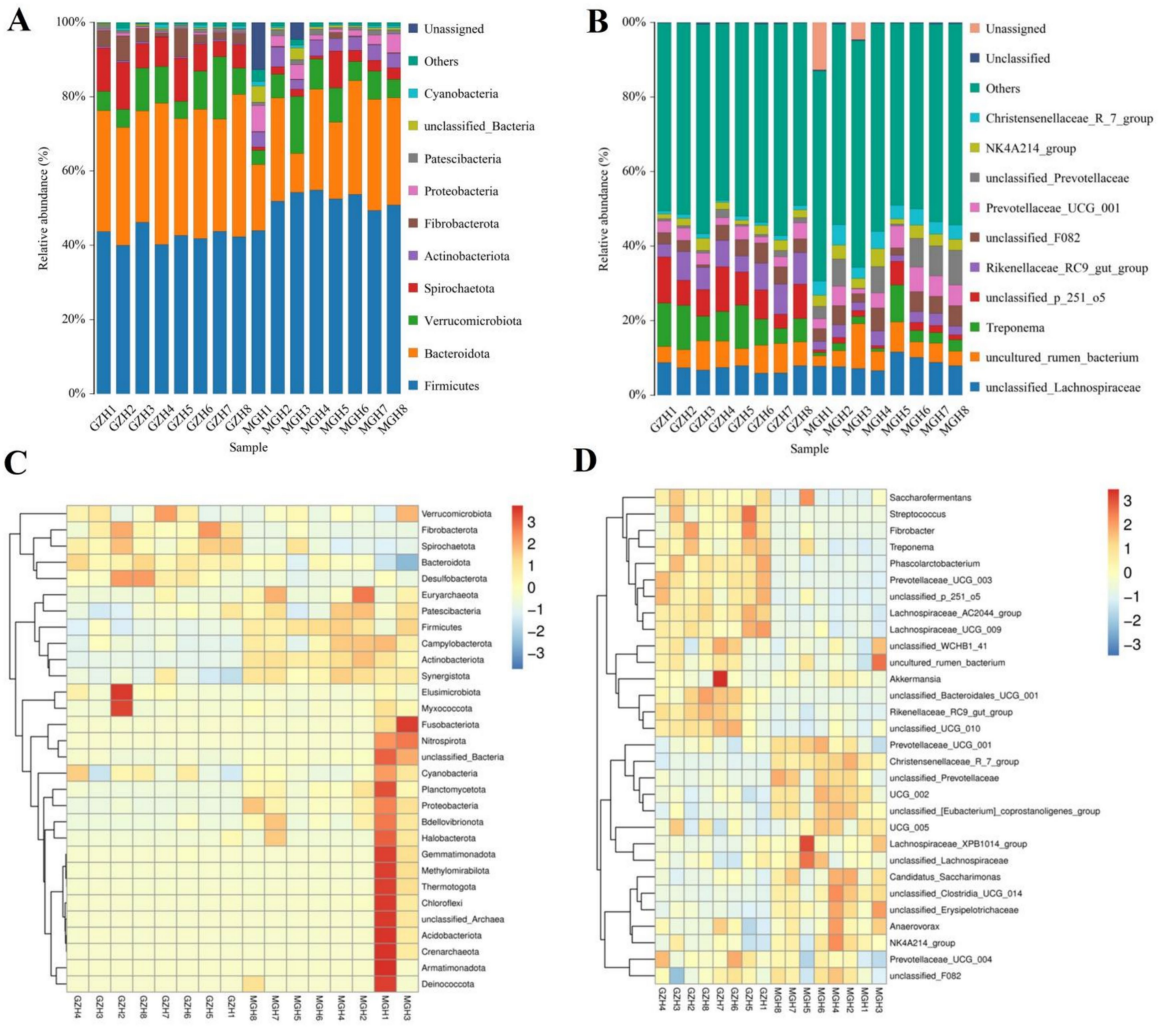
Relative abundance of the major bacterial phyla **(A)** and genera **(B)** in the samples from the MGH and GZH. Heat map showing the relative richness of the more bacterial phyla **(C)** and genera **(D)** observed in the samples from MGH and GZH. The relative abundance of bacterial phyla and genera is indicated by different colored bars.

At the phylum level, Firmicutes (42.61, 51.31%), Bacteroidota (33.35, 24.25%) and Verrucomicrobiota (8.78, 7.42%) were the most preponderant in the GZH and MGH (Figure 3A). Furthermore, other phylum such as Fibrobacterota (3.79, 0.34%), Proteobacteria (0.36, 3.15%), Patescibacteria (0.81, 1.15%), unclassified_Bacteria (0.23, 1.34%) and Cyanobacteria (0.46, 0.57%) in GZH and MGH were recognized in low abundances. Besides this, we also analyzed the species and abundance of dominant gut bacterial genera at different levels. Results indicated that a total of 771 genera were detected in 16 samples from MGH and GZH. Specifically, *unclassified_p_251_o5* (8.44%) was the most dominant bacterial genus in the GZH, followed by *Treponema* (8.30%), and *unclassified_Lachnospiraceae* (7.24%) (Figure 3B). Meanwhile, the predominant bacterial genera found in the MGH were *unclassified_Lachnospiraceae* (8.40%), *uncultured_rumen_bacterium* (5.58%) and *unclassified_Prevotellaceae* (5.50%). The abundances of more bacterial phyla and genera was also visualized via clustered heatmaps (Figures 3C,D).

**Figure 4:**
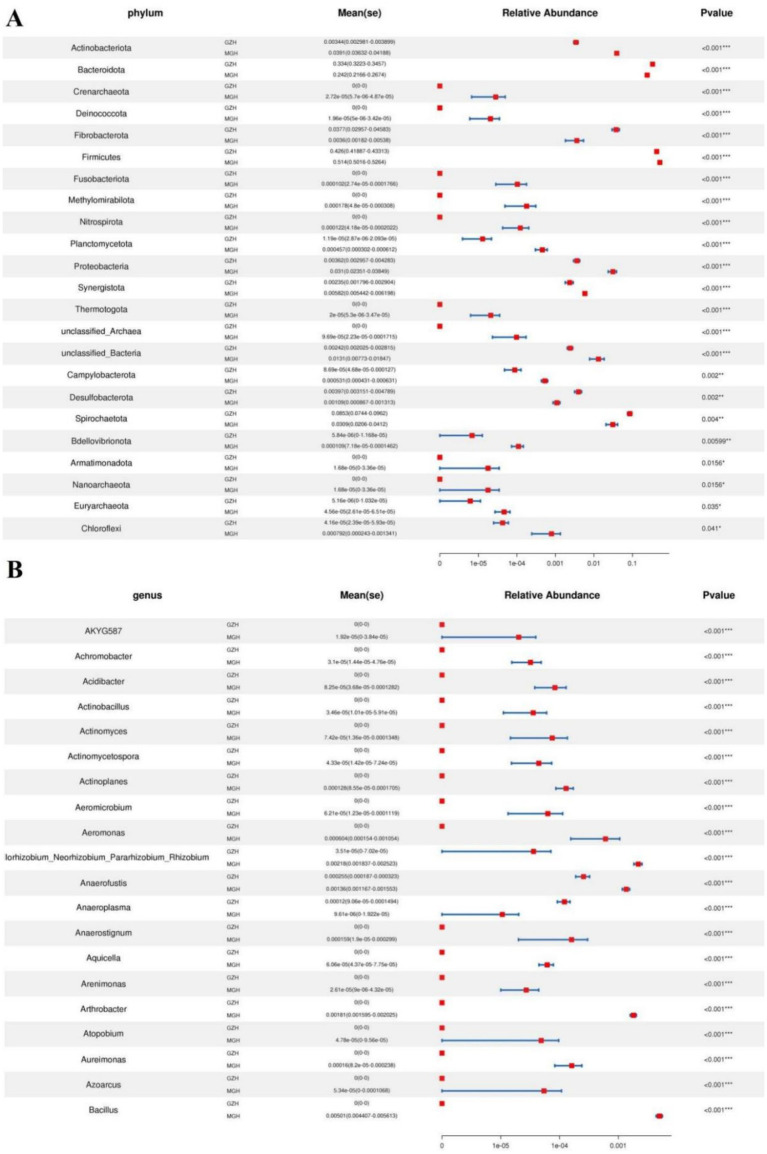
Bacteria with significant differences in relative abundance at phylum **(A)** and genus **(B)** levels between MGH and GZH. Not all data are shown. Data are expressed as the Mean ± SD. *p < 0.05, **p < 0.01, ***p < 0.001.

**Figure 5:**
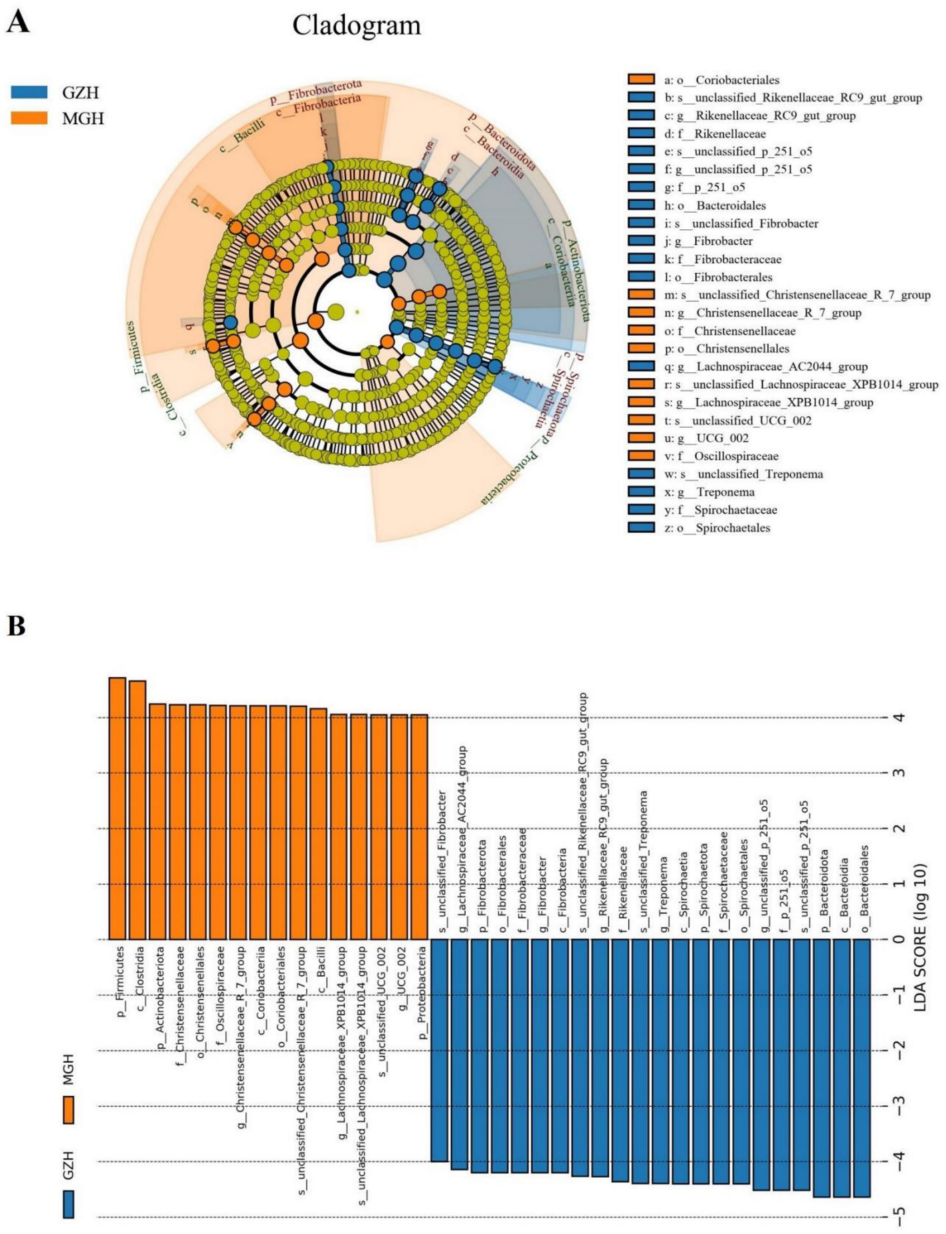
The cladogram shows significantly different taxa at five levels. **(A)** Orange circles and blue circles represent taxa that were significantly enriched in the MGH and GZH, respectively. Taxa that are not significantly different are represented by yellow circles. **(B)** Bacterial taxa with LDA greater than 4 were displayed.

Metastats analysis was conducted for recognizing bacterial taxa with distinct differences between GZH and MGH. At the phylum level, Actinobacteriota, Crenarchaeota, Deinococcota, Firmicutes, Fusobacteriota, Methylomirabilota, Nitrospirota, Planctomycetota, Proteobacteria, Synergistota, Thermotogota, unclassified_Archaea, unclassified_Bacteria, Campylobacterota, Bdellovibrionota, Armatimonadota, Nanoarchaeota, Euryarchaeota, and Chloroflexi were significantly more preponderant in the MGH than in the GZH, whereas the Bacteroidota, Fibrobacterota, Desulfobacterota, and Spirochaetota were lower (Figure 4A). Moreover, 383 bacterial genera were demonstrated to be significantly different between MGH and GZH (Figure 4B). Specifically, the relative abundances of 70 bacterial genera (*Cellulosilyticum*, *Prevotellaceae_UCG_003*, *Prevotella*, *Lachnospiraceae_AC2044_group*, *Lachnospiraceae_NK4B4_group*, *Lachnospiraceae_UCG_009*, *Rikenellaceae_RC9_gut_group*, *Roseburia*, *Weissella*, *Oscillospira*, *Ruminococcus*, etc) significantly decreased, while the relative abundances of 313 bacterial genera (*Bacillus*, *Ligilactobacillus*, *Christensenellaceae_R_7_group*, *Lactococcus*, *Prevotella_7*, *Prevotellaceae_NK3B31_group*, *Prevotellaceae_UCG_001*, *Lachnospiraceae_NK3A20_group*, *Lachnospiraceae_NK4A136_group*, *Butyricicoccus*, *Coprococcus*, *Lachnospiraceae_UCG_010*, *Lachnospiraceae_XPB1014_group*, *Ruminiclostridium*, *Eubacterium*, etc.) significantly increased in MGH as compared to GZH. Results of LEfSe analysis showed that *Christensenellaceae_R_7_group*, *Lachnospiraceae_XPB1014_group*, and *UCG_002* were the most dominant in the MGH, while *Lachnospiraceae_AC2044_group*, *Fibrobacter*, *Rikenellaceae_RC9_gut_group*, *Treponema*, and *unclassified_p_251_o5* were significantly overrepresented in the GZH (Figures 5A,B).

### Analysis of gut fungal composition and differential taxa

**Figure 6:**
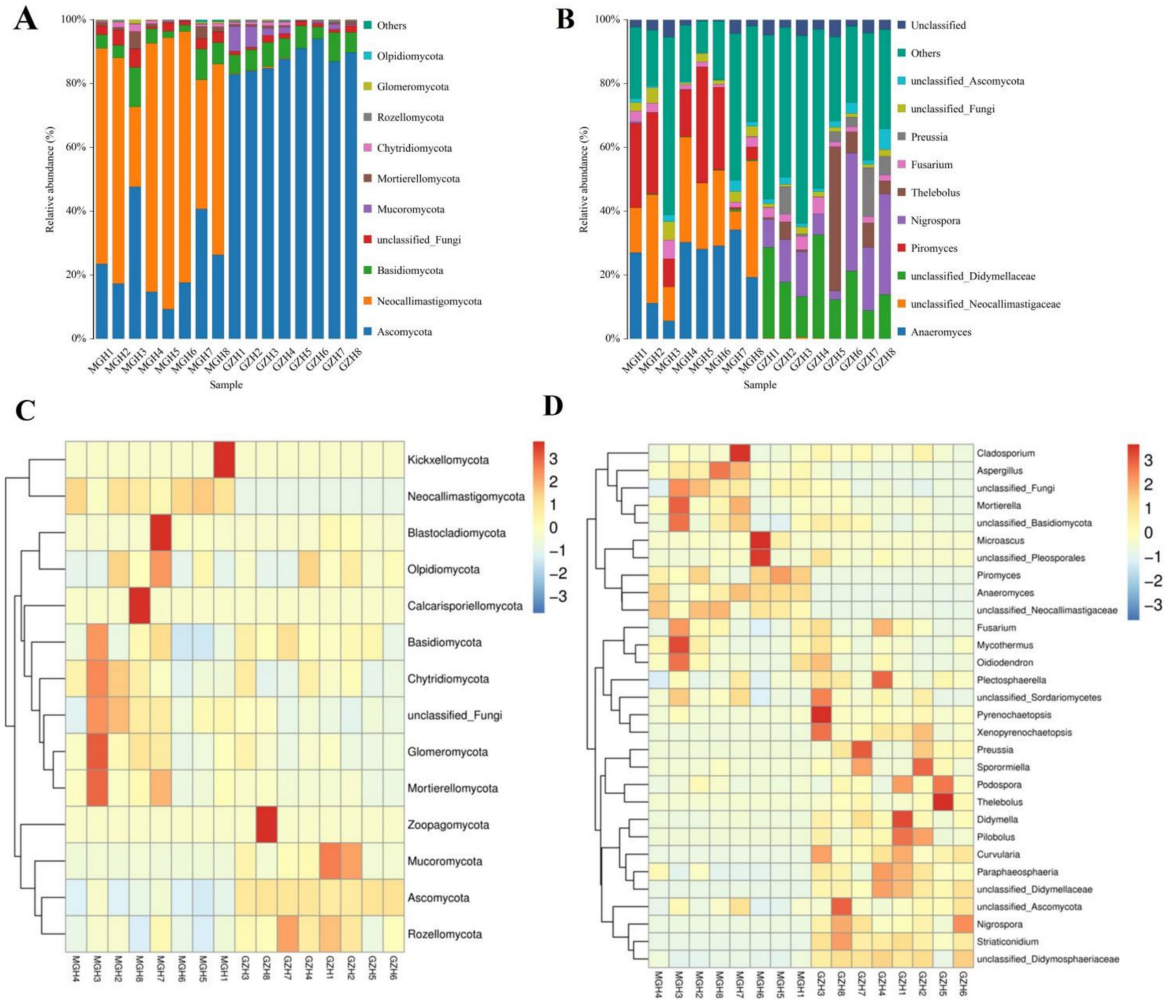
Relative abundance of major fungal phyla **(A)** and genera **(B)** in samples from the MGH and GZH. Heat map showing the relative richness of the more fungal phyla **(C)** and genera **(D)** observed in samples from MGH and GZH. The relative abundance of fungal phyla and genera is indicated by different colored bars.

At the phylum level, the gut fungal community in MGH were predominated by Neocallimastigomycota (63.98%), Ascomycota (24.09%), and Basidiomycota (5.55%) in descending order (Figure 6A). Moreover, the phyla Ascomycota (87.52%), Basidiomycota (6.59%) and Mucoromycota (2.69%) were abundantly present in GZH. Conversely, other phylum such as Chytridiomycota (0.90, 0.52%), Rozellomycota (0.12, 0.40%), Glomeromycota (0.37, 0.13%) and Olpidiomycota (0.079, 0.076%) in the GZH and MGH were identified in low abundances, which the average richness is less than 1%. Besides the fungal phylum, the abundance of fungal genera in GZH and MGH were also explored and 545 fungal genera were totally identified. Among them, *Anaeromyces* (23.06%) was the most predominant fungal genus in the MGH, followed by *unclassified_Neocallimastigaceae* (22.51%) and *Piromyces* (18.39%) (Figure 6B). Moreover, the *unclassified_Didymellaceae* (18.55%), *Nigrospora* (15.67%), and *Thelebolus* (8.52%) were abundantly present in the GZH. The abundance of more fungal phyla and genera is presented through clustered heatmaps (Figures 6C,D).

**Figure 7:**
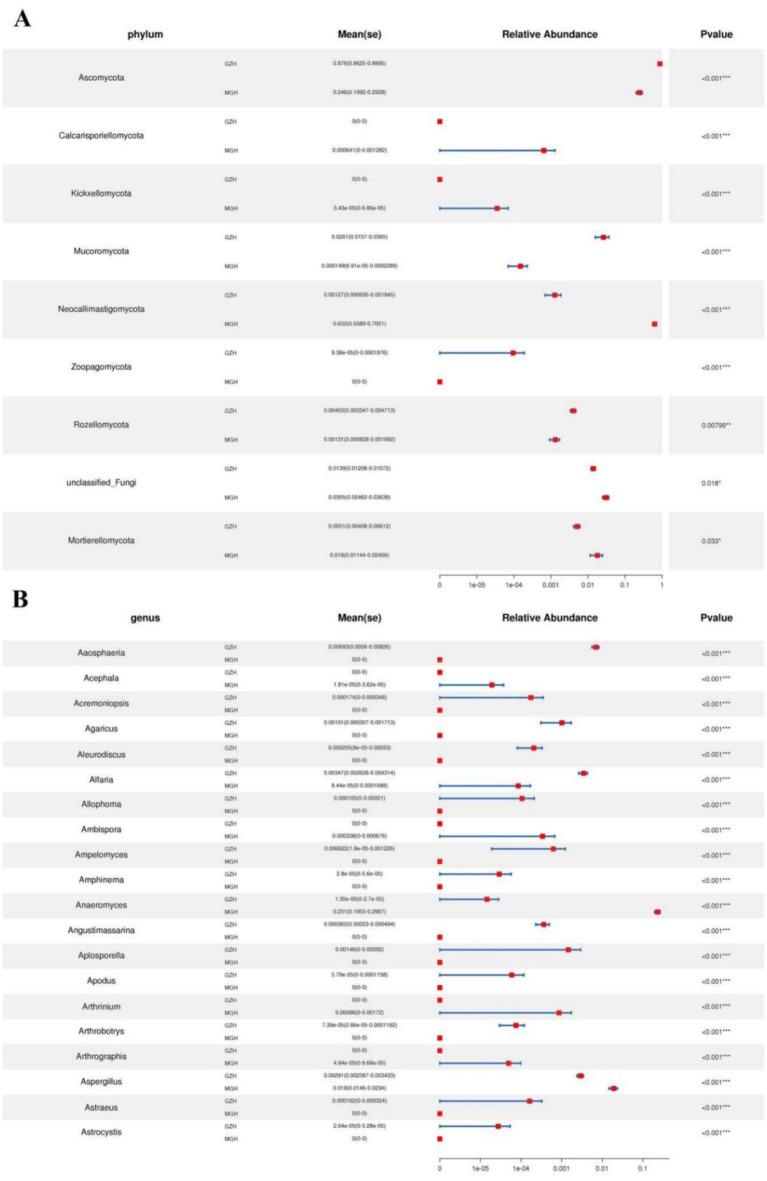
Fungi with significant differences in relative abundance at phylum **(A)** and genus **(B)** levels between MGH and GZH. Not all data are shown. Data are expressed as the Mean ± SD. *p < 0.05, **p < 0.01, ***p < 0.001.

**Figure 8:**
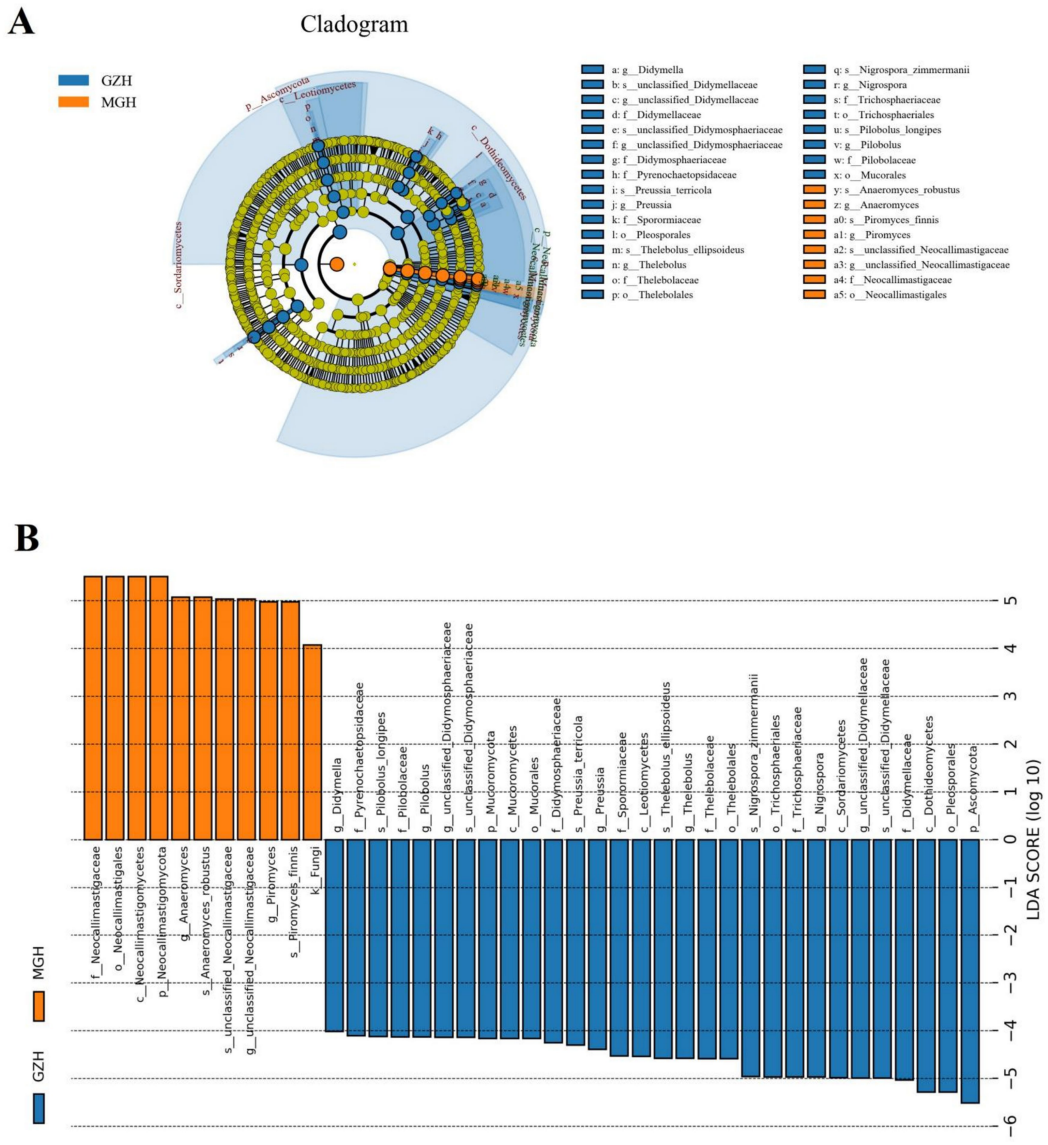
The cladogram shows significantly different taxa at five levels. **(A)** Orange circles and blue circles represent taxa that were significantly enriched in the MGH and GZH, respectively. Taxa that are not significantly different are represented by yellow circles. **(B)** Fungal taxa with LDA greater than 4 were displayed.

Metastats analysis was used to identify taxa with distinct differences between GZH and MGH. At the phylum level, the gut fungal community in the MGH showed an obvious increase in the relative abundances of Calcarisporiellomycota, Kickxellomycota, Neocallimastigomycota, unclassified_Fungi and Mortierellomycota, while Ascomycota, Mucoromycota, Zoopagomycota and Rozellomycota decreased dramatically compared with GZH (Figure 7A). Additionally, we also observed that 335 fungal genera were significantly different between the MGH and GZH (Figure 7B). Among them, the relative abundances of 215 fungal genera (*Acremoniopsis*, *Agaricus*, *Bovista*, *Buckleyzyma*, *Clypeosphaeria*, *Daldinia*, *Eleutherascus*, *Falciformispora*, *Favolus*, *Gliomastix*, *Goffeauzyma*, *Hansfordia*, *Heterocephalacria*, etc.) dramatically decreased, whereas the relative abundances of 120 fungal genera (*Acephala*, *Ambispora*, *Clathrosphaerina*, *Coniella*, *Diaporthe*, *Entrophospora*, *Floccularia*, *Geastrum*, *Geminibasidium*, *Lactifluus*, *Lambertella*, *Monosporascus*, *Ophiobolus*, etc.) decreased increased in MGH compared with GZH. LEfSe analysis indicated that the GZH was dramatically enriched for *Didymella*, *Pilobolus*, *unclassified_Didymosphaeriaceae*, *Preussia*, *Thelebolus*, *Nigrospora*, and *unclassified_Didymellaceae*, while the MGH showed a dramatically higher abundance of *Anaeromyces*, *unclassified_Neocallimastigaceae*, and *Piromyces* (Figures 8A,B).

### Correlation network analysis of gut bacterial and fungal communities

**Figure 9:**
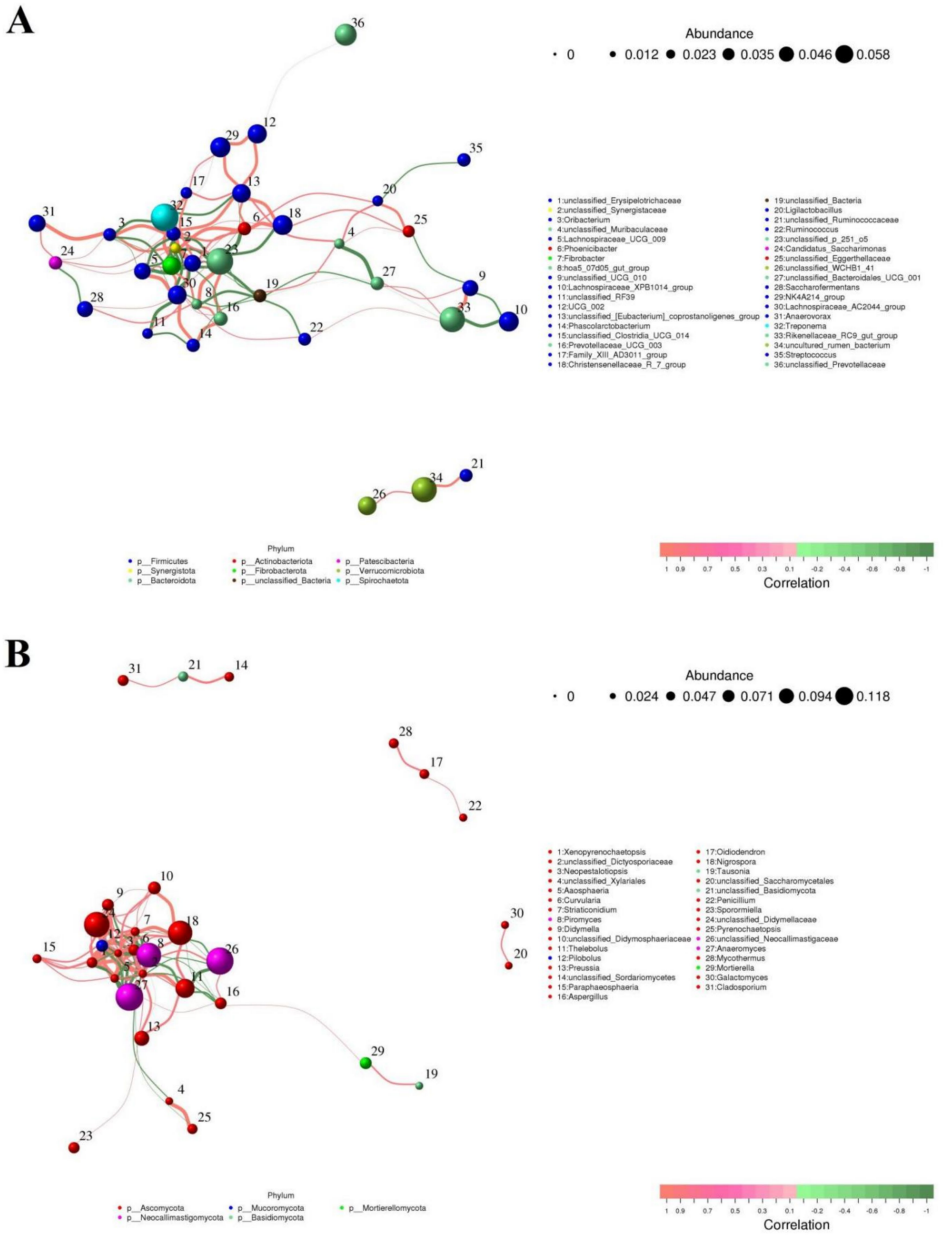
Visualization of correlations between different gut bacterial **(A)** or fungal **(B)** genera. The color (orange: positive correlation; green: negative correlation) of the lines between bacterial or fungal genera determines their correlation.

To further investigate the relationship between gut microbiota, we performed correlation analysis on some representative bacteria and fungi and drew a network interaction diagram (Figures 9A,B). In the gut bacterial community, we observed that *Lachnospiraceae_UCG_009* was positively related to *Fibrobacter* (0.82), *Lachnospiraceae_AC2044_group* (0.91) and *Treponema* (0.80), but was negatively associated with *unclassified_Erysipelotrichaceae* (−0.9). *Ligilactobacillus* was positively related to *unclassified_Eggerthellaceae* (0.86) and *Phoenicibacter* (0.83), but was negatively associated with *Streptococcus* (−0.83). *Prevotellaceae_UCG_003* was positively related to *unclassified_p_251_o5* (0.87) and *Lachnospiraceae_AC2044_group* (0.80). In the gut fungal community, we observed that *Xenopyrenochaetopsis* was positively associated with *Pilobolus* (0.95), *Curvularia* (0.86), *unclassified_Didymellaceae* (0.83), *Didymella* (0.79), but was negatively associated with *Piromyces* (−0.83). *Neopestalotiopsis* was positively associated with *Xenopyrenochaetopsis* (0.92), *Aaosphaeria* (0.91), *Pilobolus* (0.87), *Pilobolus* (0.87), *Curvularia* (0.84), *unclassified_Didymellaceae* (0.83), *unclassified_Dictyosporiaceae* (0.83), *unclassified_Didymosphaeriaceae* (0.82), *Nigrospora* (0.82), *Preussia* (0.78), *Preussia* (0.78), and *Thelebolus* (0.77), but was negatively associated with *Anaeromyces* (−0.85) and *Piromyces* (−0.85).

### Functional analysis of gut bacterial community

**Figure 10:**
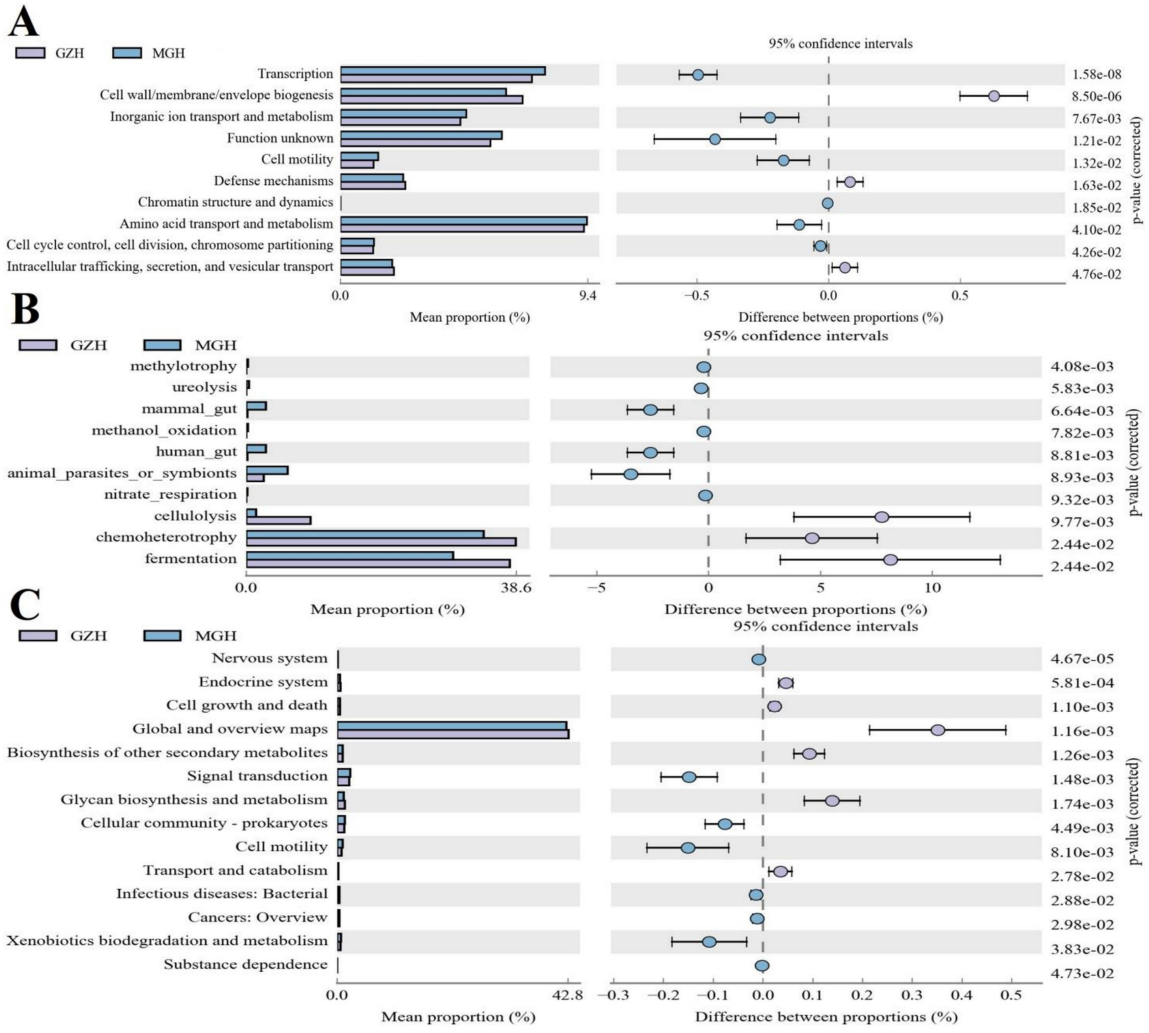
Functional prediction analysis of gut bacterial community. **(A)** COG functional prediction. **(B)** FAPROTAX functional prediction. **(C)** KEGG functional prediction.

Gut bacterial COG functional prediction analysis indicated that the relative abundances of transcription, inorganic ion transport and metabolism, cell motility, amino acid transport and metabolism, cell cycle control, cell division, chromosome partitioning, and chromatin structure and dynamics in the MGH was significantly higher than that in the GZH, while the relative abundances of cell wall/membrane/envelope biogenesis, defense mechanisms, and intracellular trafficking, secretion, and vesicular transport were lower (Figure 10A). As for the FAPROTAX functional prediction analysis, the GZH had significantly higher relative abundances of chemoheterotrophy, fermentation, and cellulolysis compared to the MGH (Figure 10B). Conversely, the relative abundances of methylotrophy and ureolysis were lower in the GZH. Results of KEGG functional prediction analysis showed that substance dependence, nervous system, signal transduction, cellular community-prokaryotes, infectious diseases: bacterial, cancers: overview, cell motility, and xenobiotics biodegradation and metabolism were significantly enriched in the MGH, whereas transport and catabolism, glycan biosynthesis and metabolism, biosynthesis of other secondary metabolites, global and overview maps, endocrine system and cell growth and death were found to be more abundant in the GZH (Figure 10C).

## Discussion

The gut microbiota is the most complex microbial ecosystem, mainly involving bacteria and fungi, which play key roles in multiple physiological functions including metabolism, immunity, and digestive absorption (Halsey et al., 2023; Li et al., 2024). Furthermore, some studies associated with gut microbiota have also revealed its important role in host traits, growth and development (Wang et al., 2022). However, the gut microbial composition is easily influenced by external factors such as environment and diet (Barber et al., 2023; Hu et al., 2024). Notably, some intrinsic factors including gender, age, and species could also affect the gut microbiota and even play an important role in many traits of animals (Lee et al., 2023; Yoon and Kim, 2021). For instance, research has indicated that there were significant differences in the gut fungal composition between the Mongolian and Dutch Warmblood horses (Lan et al., 2024). In addition, Chang et al. performed a comparative analysis of four breeds of sheep and observed that the gut microbial diversity of Tibetan sheep was significantly lower than that of Dorper, Dorset, and Small Tail Han sheep (Chang et al., 2020). These results indicated that breed has a significant impact on the gut microbiota. Horses are important livestock animals with many different breeds and functions. GZH and MGH are both ancient breeds in China, which are closely associated with local ethnic culture and social development. However, little is known about the characteristics and differences of gut microbiota between GZH and MGH. Thus, we characterized the composition and variability of gut microbiota between GZH and MGH. The results showed a significant difference in gut bacterial and fungal compositions, diversities, and structures between GZH and MGH.

The alpha and beta diversity indices are important indicators for evaluating the gut microbial diversity, abundance, and structure (Hernandez et al., 2024). Typically, Chao1 and ACE indices are used to evaluate the gut microbial abundance, while Shannon and Simpson indices are used for assessing the gut microbial diversity. Numberous studies have indicated that the gut microbial diversity and abundance are important indicators for assessing gut microbial homeostasis (Ellegaard and Engel, 2019; Li et al., 2016). However, these gut microbial diversity indices are dynamically changing due to multiple internal and external factors. Previous studies have indicated that the higher gut microbial diversity contribute to performing complex intestinal functions such as metabolism, digestion, and absorption (Shang et al., 2021; Zheng et al., 2019). Tibetan pigs inhabiting the Qinghai-Tibet Plateau possess more rich and diverse gut microbiota compared with the Diannan small ear pigs. The Qinghai-Tibet Plateau, situated over 3,500 meters above sea level, is characterized by hypoxia, harsh environment and food shortages (Guan et al., 2023). Thus, tibetan pigs need to evolve more diverse and rich gut microbiota to resist disease and achieve nutritional needs. The harsh environment and complex diet of the Qinghai-Tibet Plateau may be one of the reasons for the greater gut microbial diversity in Tibetan pigs. In this study, we observed that the diversity of gut bacterial and fungal communities in GZH was higher than that in MGH. Previous investigations have demonstrated that diet can affect the gut microbial composition and structure (Nogal et al., 2021). Compared with Mongolia, Guizhou has relatively scarce forage resources. Therefore, GZH need to evolve a more complex gut microbiota to improve intestinal function and meet nutritional and energy requirements. Moreover, we also compared the differences in the gut microbial structure between GZH and MGH. Consistent with the results of alpha diversity, beta analysis results indicated that the structure of the gut bacterial and fungal communities between GZH and MGH was significantly different. These results all demonstrated significant differences in the diversity and structure of the gut bacterial and fungal communities between GZH and MGH.

Our results indicated that *Firmicutes*, *Bacteroidota*, *Ascomycota*, and *Basidiomycota* were the most predominant taxa in the gut bacterial or fungal communities, regardless of the breed. Notably, these bacterial and fungal phyla were also abundantly present in other mammals such as cattle, yak and sheep (Chen et al., 2022; Li D. et al., 2023; Liu W. et al., 2021). Moreover, these bacterial and fungal phyla are also recognized as the most important characteristic of the mammalian gut microbiota. Although the main dominant bacterial and fungal phyla remained consistent, the abundances of some bacterial and fungal genera changed significantly between GZH and MGH. Among these differential bacteria or fungi are considered to be potential beneficial bacteria in the intestine, playing an important role in intestinal function and host health. For instance, the GZH was significantly enriched for *Cellulosilyticum*, *Prevotellaceae_UCG_003*, *Prevotella*, *Lachnospiraceae_AC2044_group*, *Lachnospiraceae_NK4B4_group*, *Lachnospiraceae_UCG_009*, *Rikenellaceae_RC9_gut_group*, *Roseburia*, *Weissella*, *Oscillospira*, and *Ruminococcus*, whereas the MGH showed a significantly higher abundance of *Bacillus*, *Ligilactobacillus*, *Christensenellaceae_R_7_group*, *Lactococcus*, *Prevotella_7*, *Prevotellaceae_NK3B31_group*, *Prevotellaceae_UCG_001*, *Butyricicoccus*, *Lachnospiraceae_NK3A20_group*, *Lachnospiraceae_NK4A136_group*, *Lachnospiraceae_UCG_010*, *Lachnospiraceae_XPB1014_group*, *Ruminiclostridium*, *Coprococcus*, and *Eubacterium*. *Bacillus* is usually exist in mammalian gastrointestinal tract and environment, displaying multiple health benefits. *Bacillus could* secrete antimicrobial peptide and vitamin, showing a positive effect to intestinal health and resisting bacterial infection (Liu S. et al., 2021; Puan et al., 2023; Tang et al., 2020). Moreover, it could also maintain gut microbial balance and improve intestinal permeability (Gao et al., 2024; Yuan et al., 2024). *Cellulosilyticum* has long been regarded as potential beneficial bacteria, due to its ability to digest and decompose carbohydrate, pectin and cellulose (Miller et al., 2011). Several studies involving *Ligilactobacillus* have revealed its important roles in resisting bacterial infection, maintaining growth performance and improving immunity (Lee et al., 2022; Yang et al., 2023). *Christensenellaceae* has been reported to involved in the positive regulation of the healthy homeostasis, intestinal environment and immunity (Waters and Ley, 2019). Moreover, *Christensenellaceae* could produce digestive enzyme associated with the feed efficiency such as *β*-glucosidase, *α*-arabinosidase and β-galactosidase (Guan et al., 2024). *Lactococcus* was vital intestinal probiotics, which could improve host immunity, metabolism, and digestion (Wang et al., 2024; Zhai et al., 2023). Moreover, *Lactococcus* has been demonstrated to maintain gut microbial homeostasis and secrete antimicrobial peptides (Chen G. et al., 2021). It has been demonstrated that *Prevotella* and *Prevotellaceae* has greater metabolic diversity, which play key roles in carbohydrate metabolism including starch, hemicellulose and xylan (Bandarupalli and St-Pierre, 2020; Emerson and Weimer, 2017). *Butyricicoccus* and *Lachnospiraceae* have been demonstrated to negatively correlated with intestinal inflammation (Huang et al., 2023; Steppe et al., 2014). *Rikenellaceae* was previously reported to alleviate inflammation and degrade plant derived polysaccharide (Chen F. et al., 2021). Recent investigation on *Ruminiclostridium* has provided evidence of its important roles in promoting animal growth and decreasing gastrointestinal diseases (Vita et al., 2018). *Weissella* was regarded as potential probiotics because of its antioxidant, anti-inflammatory, and anti-disease effects (Aburas et al., 2020; Ahmed et al., 2022). Moreover, it also showed significant potential in improving the growth performance and maintaining gut microbial balance (Liu et al., 2024; Yan et al., 2023). Interestingly, several members of above-mentioned bacteria such as *Roseburia*, *Oscillospira*, *Ruminococcus*, *Ruminiclostridium*, *Coprococcus*, and *Eubacterium* were demonstrated to produce short-chain fatty acids (SCFAs). SCFAs are a group of metabolites that are beneficial to host health and have multiple functions such as alleviating oxidative stress, improving intestinal environment and inhibiting opportunistic pathogens (Abdalkareem et al., 2022; Saikachain et al., 2023). These results indicated that although there were significant differences in the gut microbial composition between GZH and MGH, all of these for achieving complicated intestinal functions and intestinal functions diversity.

Previous studies indicated that gut microbiota including gut bacterial and fungal communities could cooperate with each other in a synergistic or antagonistic manner to gut intestinal microbial homeostasis (Li et al., 2022; Scarpellini and Rinninella, 2023). Thus, we also performed correlation network analysis to explore the interactions between different gut bacterial or fungal communities. Results indicated that some functional bacteria or fungi can affect each other in multiple ways, which may contribute to further maintaining the gut microbial homeostasis and conduct intestinal functions. Furthermore, to further explore the differences in intestinal functions between MGH and GZH, we also conducted functional predictions for bacteria with significant difference. Results indicated that GZH have stronger fermentation, and cellulolysis capabilities. We speculated that environmental or dietary factors caused GZH to evolve more complicated gut microbiota and intestinal functions to adapt to their energy and nutritional needs during growth.

## Conclusion

Taken together, this study revealed the significant differences in the gut bacterial and fungal communities between MGH and GZH. Results showed that the differences between MGH and GZH were mainly manifested in the diversity and abundance of gut bacterial and fungal communities. Moreover, we also found significant differences in the abundance of some bacterial and fungal phyla and genera between the MGH and GZH. Notably, this study also highlighted the presence of specific microbial genera and metabolic functions in MGH or GZH, which may be the result of the evolution of different horse breeds to adapt to local environments and diets. However, some limitations in this study need to be mentioned including the small sample size and the lack of metabolomic analysis.

## Data Availability

The original sequence data was submitted to the Sequence Read Archive (SRA) (https://www.ncbi.nlm.nih.gov/sra) with the accession no. PRJNA1127030.
